# Biotinidase deficiency: Genotype-biochemical phenotype association in Brazilian patients

**DOI:** 10.1371/journal.pone.0177503

**Published:** 2017-05-12

**Authors:** Taciane Borsatto, Fernanda Sperb-Ludwig, Samyra E. Lima, Maria R. S. Carvalho, Pablo A. S. Fonseca, José S. Camelo, Erlane M. Ribeiro, Paula F. V. de Medeiros, Charles M. Lourenço, Carolina F. M. de Souza, Raquel Boy, Têmis M. Félix, Camila M. Bittar, Louise L. C. Pinto, Eurico C. Neto, Henk J. Blom, Ida V. D. Schwartz

**Affiliations:** 1 Post Graduate Program in Genetics and Molecular Biology, Universidade Federal do Rio Grande do Sul (UFRGS), Porto Alegre, RS, Brazil; 2 BRAIN Laboratory, Center for Experimental Research (CPE), Hospital de Clínicas de Porto Alegre (HCPA), Porto Alegre, RS, Brazil; 3 Centro Universitário Ritter dos Reis, Porto Alegre, RS, Brazil; 4 Universidade Federal de Minas Gerais, Belo Horizonte, MG, Brazil; 5 Hospital das Clínicas da Faculdade de Medicina de Ribeirão Preto, Universidade de São Paulo, Ribeirão Preto, SP, Brazil; 6 Hospital Infantil Albert Sabin, Fortaleza, CE, Brazil; 7 Universidade Federal de Campina Grande, Campina Grande, PB, Brazil; 8 Medical Genetics Service, HCPA, Porto Alegre, RS, Brazil; 9 Departamento de Pediatria, Universidade do Estado do Rio de Janeiro, Rio de Janeiro, RJ, Brazil; 10 Hospital Infantil Joana de Gusmão, Florianópolis, SC, Brazil; 11 CTN Diagnósticos, Porto Alegre, RS, Brazil; 12 Laboratory of Clinical Biochemistry and Metabolism, Department of General Pediatrics, Adolescent Medicine and Neonatology, University Medical Centre Freiburg, Freiburg, Germany; Hospital Israelita Albert Einstein, BRAZIL

## Abstract

**Introduction:**

The association between the *BTD* genotype and biochemical phenotype [profound biotinidase deficiency (BD), partial BD or heterozygous activity] is not always consistent. This study aimed to investigate the genotype-biochemical phenotype association in patients with low biotinidase activity.

**Methods:**

All exons, the 5'UTR and the promoter of the *BTD* gene were sequenced in 72 Brazilian individuals who exhibited low biotinidase activity. For each patient, the expected biochemical phenotype based on the known genotype was compared with the observed biochemical phenotype. Additional non-genetic factors that could affect the biotinidase activity were also analysed.

**Results:**

Most individuals were identified by neonatal screening (n = 66/72). When consecutive results for the same patient were compared, age, prematurity and neonatal jaundice appeared to affect the level of biotinidase activity. The biochemical phenotype at the time of the second blood collection changed in 11/22 patients compared to results from the first sample. Three novel variants were found: c.1337T>C (p.L446P), c.1466A>G (p.N489S) and c.962G>A (p.W321*). Some patients with the same genotype presented different biochemical phenotypes. The expected and observed biochemical phenotypes agreed in 68.5% of cases (concordant patients). The non-coding variants c.-183G>A, c.-315A>G and c.-514C>T were present in heterozygosis in 5/17 discordant patients. In addition, c.-183G>A and c.-514C>T were also present in 10/37 concordant patients.

**Conclusions:**

The variants found in the promoter region do not appear to have a strong impact on biotinidase activity. Since there is a disparity between the *BTD* genotype and biochemical phenotype, and biotinidase activity may be affected by both genetic and non-genetic factors, we suggest that the diagnosis of BD should be based on more than one measurement of plasma biotinidase activity. DNA analysis can be of additional relevance to differentiate between partial BD and heterozygosity.

## Introduction

Biotinidase (EC 3.5.1.12) plays a critical role in the absorption of biotin from dietary sources and in biotin recycling. In biotinidase deficiency (BD, OMIM: 253260), an autosomal recessive disease caused by pathogenic mutations in the *BTD* gene, both deficiencies of biotin and in biotin-dependent carboxylases occur [1, 2]. Treatment with oral administration of free biotin can prevent the symptoms (cutaneous rash, seizures, motor, hearing and vision problems, developmental delay, coma, and death) and should ideally be started during the neonatal period [3].

The measurement of biotinidase activity in plasma or serum by colorimetric assay [2] is the most frequently used method for the diagnosis of BD. Unfortunately, a number of issues can influence the measurement of biotinidase activity, including the temperature of the sample during storage or transport, the presence of jaundice, prematurity and even postnatal age [4–6], resulting in erroneously lower readings. Sequencing of the *BTD* gene is an additional method used to establish the correct diagnosis and make decisions about life-long treatment, e.g., patients with profound or partial BD should receive daily oral biotin supplementation. A substantial number of severe mutations in homozygosis or compound heterozygosis are associated with profound BD (biotinidase activity <10% of average normal activity). Compound heterozygosity between c.1330G>C (p.D444H) and a severe mutation is associated with partial BD (activity within 10–30%). Individuals who are homozygous for p.D444H show plasma biotinidase activity similar to the heterozygous for a severe mutation [7]. Compound heterozygosity between one severe mutation and one of the next four less frequent mutations [c.968A>G (p.H323R), c.257T>G (p.M86R), c.310-15delT [8] and c.529G>A (p.E177K)] is likely to cause partial BD [9]. However, the association between the *BTD* genotype and biotinidase activity is not always consistent [10–13]. This disagreement may be due to the aforementioned factors that affect biotinidase activity or unknown factors, such as genetic variants in non-coding regions of the *BTD* gene.

In 2014, we published a study on the genetic evaluation of 38 Brazilian patients with low biotinidase activity [12]. The current manuscript represents an extension of that study and presents data for 34 additional patients, including analysis of the promoter region, 5'UTR and exon 1 of the *BTD* gene for all 72 patients. In addition, we investigated the relationship between genotype and biochemical phenotype.

## Methods

This is a multicentre, observational, cross-sectional study with a convenience sampling strategy that was approved by the Ethics in Research Committee of Hospital de Clínicas de Porto Alegre, Brazil. All patients and/or their legal guardians provided written informed consent.

In Brazil, neonatal screening for BD is available in private and public health systems. The public system has wide coverage, reaching more than 84% of Brazilian live births in 2014 [14]. According to the National protocol, blood must be collected on filter paper between the 3rd and 5th days of life. If the result is abnormal, a second collection and test on filter paper must be performed. If the second result is also decreased, biotin treatment should be initiated and, after 3 months, an additional confirmatory test should be performed. The confirmatory test is the quantitative measurement of biotinidase activity in plasma. If the result is profound or partial BD (activity below 30%), treatment and medical follow-up should be maintained. If the result is higher than 30%, treatment is stopped. Tests are performed in reference laboratories [15].

### Subjects

The total sample comprised 72 individuals (40 males), including one pair of siblings, aged one month to 18 years, who were recruited from 2012 to 2015 by contacting Brazilian medical geneticists through the mailing list of the Brazilian Medical Genetics Society. The inclusion criterion was reduced biotinidase activity (a value below the normal reference range for the testing laboratory, i.e., heterozygous level, partial or profound BD) detected by quantitative testing after abnormal neonatal screening (n = 59 patients) or after clinical suspicion (n = 6 patients). In addition to these participants, seven subjects (identified by neonatal screening) who had no quantitative test were also included in the study because the special interest of the families and geneticists in the molecular analysis, once they did not have access to the confirmatory test.

Clinical information and blood samples were collected from patients and their parents (samples from both parents were available for 55 patients, samples from only one parent were available for nine patients, and no parental samples were available for eight patients). The patients came from several regions of Brazil (South = 42, Southeast = 15, Northeast = 15).

### Biotinidase activity

Quantitative enzyme activity testing results were available for 65/72 patients. All laboratories (n = 11) that performed the tests used the colorimetric testing in plasma or serum described by Wolf et al. [2], and the normal reference range of 5.0–10 nmol/min/mL was applied. The mean value of 7.5 nmol/min/mL was used to classify the biochemical phenotype among patients who presented activity lower than 5.0 nmol/min/mL: <0.75 (<10%), profound BD; 0.75–2.25 (10–30%), partial BD; and 2.26–4.99 (30.1–66.5%), heterozygous activity. Patients whose biotinidase activity was 5.0–10.0 nmol/min/mL were considered to have 100% activity. Values within ± 0.1 of a cut-off point were classified as borderline. When more than one measurement was available, the highest value was used for classification purposes. For the remaining patients (n = 7/72), only qualitative enzyme activity testing results were available; thus, these patients were not included in the biochemical phenotyping.

### DNA analysis

In our previous study, the DNA of 38 patients was extracted from blood samples in EDTA, and exons 2, 3, and 4, as well as the exon/intron junctions of the *BTD* gene, were sequenced [12]. In the present study, the same analysis was performed for the additional 34 participants. Furthermore, the promoter region, 5'UTR and exon 1 (according to annotation at GenBank, accession no. AF018630.1) were analysed for all 72 samples. A fragment of 715 base pairs (bp) including the *BTD* promoter, 5’UTR, and exon 1 was amplified by PCR using the primers 5’-CCCCACCATATCGCCACATCC-3’ and 3’-CCGCACGCCCTTACCACA-5’ and an annealing temperature of 67°C, followed by Sanger sequencing. This strategy allowed for analysis of the 599 bp upstream of the first translation start site. Parental samples were assessed to define whether the identified variants were in *cis* or *trans*. The sequence NG_008019.1 was used as the reference sequence for the *BTD* gene.

Since the sample included one pair of siblings and five children of consanguineous parents, the total number of alleles was 137.

The establishment of the expected biochemical phenotype to each genotype found was based on the literature [7]. As a result, we could only include genotypes composed of variants with known clinical significance (reported at ARUP database, http://utahedu.aruplab.com/database/BTD/BTD_welcome.php). Non-coding variants were not considered in this classification.

A portion of exon 4 of the *BTD* gene (exon 4d) was sequenced in anonymous samples from 100 healthy, adult controls from Southern Brazil as part of our previous study [12]. Those results were reassessed to verify the frequencies of the missense variants described for the first time in the present study, c.1337T>C or p.L446P and c.1466A>G or p.N489S.

The frequency of the p.D444H allele and the frequency of patients classified as partial BD based on enzyme activity and genotype were compared between South, Southeast and Northeast Brazil using the Chi-square test. The significance level of 5% was adopted and IBM SPSS Statistics for Windows version 23.0 software (Armonk, NY: IBM Corp) was used.

### Comparison between genotype and biochemical phenotype

For each genotype observed, the biochemical phenotype (profound BD, partial BD, heterozygous activity, normal activity) presented by the patients was verified. Furthermore, the expected biochemical phenotype (based on the genotype) and the observed biochemical phenotype were compared in patients for whom both classifications were available, and these comparative results were classified as being either concordant or discordant. When the biotinidase activity was borderline between two categories (e.g., between profound and partial BD), the expected biochemical phenotype was considered concordant if one of the two categories was consistent with the expected biochemical phenotype.

### Bioinformatics analysis

The pathogenicity of the novel variants p.L446P and p.N489S was evaluated using PolyPhen-2 [16], SIFT [17] and Mutation Taster [18] software.

Conservation in the region surrounding the non-coding variants in the *BTD* promoter (chr3:15637844–15648175 according to GRCh37/hg19) was evaluated using ECR Browser [19] (http://ecrbrowser.dcode.org/). Evolutionary conservation of the nucleotides at the non-coding variant positions was then assessed using the multiple alignment tool MAFFT version 7 [20] (http://mafft.cbrc.jp/alignment/server/). The Alibaba version 2.1 tool was used to predict transcription factor binding sites that could be created or abolished by the non-coding variants (http://www.gene-regulation.com/pub/programs.html). The Gene Cards database [21] (www.genecards.org) was consulted to compare the expression profiles (mRNA and protein) of biotinidase and transcription factors that had binding sites predicted to be created/abolished in the *BTD* gene. This comparison focused on liver and pancreas expression patterns, where serum and intestinal biotinidase is produced. Finally, the ENCODE ChIP-Seq Significance Tool [22] (http://encodeqt.simple-encode.org/) was used to verify whether the predicted binding sites for transcription factors were corroborated by the ChIP-Seq experiments.

## Results

### Clinical data and biochemical classification

Sixty-six patients were identified by neonatal screening, 55 of whom were receiving biotin supplementation at inclusion; none had clinical manifestations suggestive of BD. Six patients were preterm (35–36 weeks of pregnancy), and neonatal jaundice was reported for another six patients.

Six patients were diagnosed based on clinical suspicion. In these patients, the most common symptoms were visual disturbances, neurological manifestations, and skin lesions. The age at onset of clinical manifestations ranged from one day to 10 years, and the age at diagnosis ranged from 40 days to 18 years. All symptomatic patients were receiving biotin supplementation (10–20 mg/day) at the time of the study. Of the 71 unrelated patients, 5 (7%) had consanguineous parents; one (1.4%) had a family history suggestive of BD (the patient had an older sister who died at 3 years old with a clinical picture consistent with BD but with no biochemical or genetic diagnosis), and one (1.4%) had a confirmed family history of BD (the pair of siblings included in the study).

The biotinidase activity (quantitative assay) and observed biochemical phenotype were determined for 65/72 patients (Table 1). Four (6.1%) patients were classified as profound BD, 19 (29.2%) as partial BD, 34 (52.3%) as heterozygous, six (9.2%) as borderline activity (three between partial BD and heterozygous and three between heterozygous and normal activity), and two (3%) as abnormal activity at inclusion in the study but normal thereafter (patients with genotypes 08 and 36 in Table 1). The frequency of partial BD tended to be higher in the South Brazilian region (38.9%) than in the Southeast (20%) and Northeast (14.3%), although this was not statistically significant (exact p-value = 0.198).

**Table 1:** Biotinidase deficiency: Genotypes and biochemical phenotypes presented by 72 Brazilian patients.

Genotype	Number of patients per genotype	Allele 1^a^		Allele 2^a^		Expected biochemical phenotype^b^	Observed biochemical phenotype^C^
		cDNA change	protein change	cDNA change	protein change		
01	2	N	N	N	N	N	Hz
02	2	c.*-514C>T*	N	N	N	N	Hz
03	1	c.[*-183G>A;1413T>C*]	p.*C471C*	N	N	N	Borderline Hz/N
04	1	c.[*-183G>A;1413T>C*]	p.*C471C*	c.[*-183G>A;1413T>C*]	p.*C471C*	N	NA
05	7	c.1330G>C	p.D444H	N	N	N	Partial (n = 1), Hz (n = 4), Borderline Hz/N (n = 2)
06	1	c.1330G>C	p.D444H	c.*-183G>A*	N	N	Hz
07	1	c.1330G>C	p.D444H	c.*1284C>T*	p.*Y428Y*	N	Hz
08	16	c.1330G>C	p.D444H	c.1330G>C	p.D444H	Hz	NA (n = 5), Borderline Partial/Hz (n = 1), Hz (n = 9), N (n = 1)
09	1	c.278A>G	p.Y93C	N	N	Hz	Borderline Partial/Hz
10	1	c.643C>T	p.L215F	N	N	Hz	Hz
11	1	c.[*-183G>A*;595G>A;*1413T>C*]	p.[V199M;*C471C*]	N	N	Hz	Hz
12	1	c.1368A>C	p.Q456H	c.*-514C>T*	N	Hz	Hz
13	1	c.1595C>T	p.T532M	c.[*-514C>T;-183G>A*]	N	Hz	Hz
14	1	c.[1330G>C;511G>A]	p.[D444H;A171T]	c.[*-183G>A;1413T>C*]	p.*C471C*	Hz	Hz
15	1	c.1481A>G	p. Y494C	c.[*-183G>A;1413T>C*]	p.*C471C*	Hz	Hz
16	1	c.1330G>C	p.D444H	c. 98_104delinsTCC	p.C33fs	Partial	Partial
17	6	c.1330G>C	p.D444H	c.755A>G	p.D252G	Partial	Partial (n = 4), Hz (n = 2)
18	1	c.1330G>C	p.D444H	c.933delT	p.S311fs	Partial	Partial
19	1	c.1330G>C	p.D444H	c.1368A>C	p.Q456H	Partial	Partial
20	1	c.1330G>C	p.D444H	c.1629C>A	p.D543E	Partial	Hz
21	2	c.1330G>C	p.D444H	c.[1330G>C;470G>A]	p.[D444H;R157H]	Partial	Partial
22	3	c.1330G>C	p.D444H	c.[1330G>C;511G>A]	p.[D444H;A171T]	Partial	NA (n = 1), Partial (n = 1), Hz (n = 1)
23	1	c.1330G>C	p.D444H	c.[*1284C>T*;1489C>T]	p.[*Y428Y*;P497S]	Partial	Partial
24	2 (siblings)	c.1330G>C	p.D444H	c.[*-183G>A*;594_596delCGT]	p.V199del	Partial	Partial
25	1	c.1330G>C	p.D444H	c.[*-183G>A*;595G>A;*1413T>C*]	p.[V199M;*C471C*]	Partial	Hz
26	1	c.1612C>T	p.R538C	c.1612C>T	p.R538C	Profound	Profound
27	1	c.643C>T	p.L215F	c.755A>G	p.D252G	Profound	Profound
28	1	c.755A>G	p.D252G	c.755A>G	p.D252G	Profound	Profound
29	1	c.1227_1241delins11	p.W409fs	c.1227_1241delins11	p.W409fs	Profound	Profound
30	1	c.[1330G>C (;) 100G>A]	p.[D444H (;) splice site or G34S]	phase not confirmed		Partial or Hz	Partial
31	1	c.[1330G>C (;) 643C>T]	p.[D444H (;) L215F]	phase not confirmed		Partial or Hz	Hz
32	1	c.[1330G>C (;) 1629C>A]	p.[D444H (;) D543E]	phase not confirmed		Partial or Hz	Hz
33	2	c.[1330G>C (;) 98_104delinsTCC]	p.[D444H (;) C33fs]	phase not confirmed		Partial or Hz	Partial
34	1	c.[**962G>A** (;) *1413T>C*]	p.[W321* (;) *C471C*]	phase not confirmed		Unknown	Hz
35	1	c.664G>A	p.D222N	c.*-514C>T*	N	Unknown	Hz
36	1	**c.1337T>C**	**p.L446P**	c.[*-183G>A;1413T>C*]	p.*C471C*	Unknown	N
37	1	c.1330G>C	p.D444H	c.119T>C	p.L40P	Unknown	Partial
38	1	c.1330G>C	p.D444H	c.479G>A	p.C160Y	Unknown	Borderline Partial/Hz
39	1	c.[1330G>C;511G>A]	p.[D444H;A171T]	**c.1466A>G**	**p.N489S**	Unknown	Partial

Novel variants are in bold; non-pathogenic variants are in italics. N, normal; Hz, heterozygous; NA, not available.

^a^ For the variant c.-315A>G (not shown), the alleles presented the nucleotide G except for one patient with genotype 5 (dp7) who was heterozygous for this variant (the allele A was in *trans* with the variant c.1330G>C).

^b^ According to the literature, see Wolf (2012).

^C^ The observed biochemical phenotype is underlined when it does not agree (it is better or worse) with the expected biochemical phenotype according to genotype; see Table 2 for more details. The following enzyme activity ranges (in nmol/min/mL) were used for the classification of the biochemical phenotype (percentage in relation to the mean value of normality): <0.75 (<10%, profound deficiency); 0.75–2.25 (10–30%, partial deficiency); 2.26–4.99 (30.1–66.5%, heterozygosity). Any value in the normal range (5.0–10) was considered as corresponding to 100% of the normal activity. Values within ± 0.1 of a cut-off point were classified as borderline.

**Table 2:** Clinical information for patients with discordance between the observed and expected biochemical phenotypes (n = 17/72 patients).

	Discordant patient (patient in Table 3)	Genotype in Table 1	Premature	Neonatal jaundice	Age at enzyme activity testing	Origin of patient (region of Brazil)	Site of testing laboratory (region of Brazil)	Clinical signs	Reference
**Patients with observed biochemical phenotype worse than expected**	dp1	01	No	Yes	1m10d	Northeast	South	No	Present study
	dp2	01	No	NR	18y8m	Southeast	South	Yes	[9]
	dp3	02	No	No	3m10d	Northeast	Northeast	No	Present study
	dp4	02	No	NR	7m	Southeast	South	Yes	[9]
	dp5	05	No	NR	2m	Northeast	South	No	Present study
	dp6 (p1)	05	No	NR	18y2m; 18y7m	Southeast	South	Yes	[9]
	dp7	05	No	NR	NA	Northeast	Northeast	No	Present study
	dp8	05	No	No	NA	Northeast	South	No	Present study
	dp9	05	No	No	NA	Northeast	Southeast	No	Present study
	dp10	06	No	NR	2m	Northeast	South	No	[9]
	dp11	07	No	No	3m27d	Southeast	Southeast	No	Present study
**Patients with observed biochemical phenotype better than expected**	dp12 (p14)	08	No	No	1m17d; 1y6m	Southeast	South	No	Present study
	dp13	17	No	NR	1y3m	South	Southeast	No	[9]
	dp14 (p16)	17	No	No	2m; 2m16d; 1y	South	South	No	Present study
	dp15 (p22)	20	No	No	NA	Southeast	South	No	Present study
	dp16	22	No	NR	1y	South	Southeast	No	[9]
	dp17	25	No	NR	2m	Northeast	Southeast	No	[9]

y, years; m, months; d, days; NR, not reported; NA, not available.

Among all patients, 22 (14 male, 8 female) had more than one biotinidase activity measurement (Table 3). The median difference in age between the first and second blood collection was 70 days (range: 2 to 572 days), and the median difference in biotinidase activity was 9.3% (range: -41.3 to 82.7%). The classification of 11 (9 male, 2 female) patients changed to a better or worse enzyme activity classification between consecutive tests. The changes occurred between the first and second blood collection, with most showing improvement, i.e., moving from a more to a less severe category (n = 8/11). Prematurity and jaundice were present only in the group of patients who moved to a better classification.

**Table 3:** Clinical information of patients with more than one biotinidase activity measurement (n = 22/72 patients).

	Patient	Genotype in Table 1	Sex	Prematurity	Neonatal jaundice	1^st^ collection			2^nd^ collection			3^rd^ collection		
						Age	Lab	Result	Age	Lab	Result	Age	Lab	Result
**1) Patients with the same classification over time**	p1	05	F	No	NR	18y2m	2	Hz (50.6%)	18y7m	2	Hz (36%)			
	p2	08	M	No	No	1m7d	1	Hz (37.3%)	1m14d	1	Hz (36%)			
	p3	08	M	No	No	29d	1	Borderline Partial/Hz (29.3%)	1m1d	2	Hz (40%)			
	p4	10	F	No	No	11d	1	Borderline Partial/Hz (29.3%)	1m	2	Hz (45.3%)			
	p5	17	F	No	No	1m5d	1	Partial (14.6%)	3m22d	2	Partial (24%)	1y5m	2	Partial (25.3%)
	p6	21	M	No	No	2m5d	1	Partial (25.3%)	5m26d	2	Partial (25.3%)			
	p7	24	F	No	NR	15d	1	Partial (16%)	22d	1	Partial (25.3%)			
	p8	28	M	No	NR	2m23d	2	Profound (1.3%)	3m	2	Profound (5.3%)	2y7m	2	Profound (4%)
	p9	30	F	No	NR	14d	1	Partial (16%)	7m	2	Partial (26.6%)			
	p10	33	M	No	no	24d	NA	Partial (21.3%)	2m13d	NA	Partial (26.6%)	6m21d	NA	Partial (24%)
	p11	39	F	No	no	1m24d	1	Borderline Profound/Partial (9.3%)	5m15d	2	Partial (18.6%)			
**2) Patients with different classifications over time**														
**2a) from a more to a less severe category**	p12	03	M	Yes	NR	1m1d	1	Profound (1.3%)	1m24d	2	Hz (34.6%)	11m27d	2	Borderline Hz/N (65.3%)
	p13	08	M	No	NR	1m22d	1	Borderline Profound/Partial (9.3%)	NA	2	Hz (44%)			
	p14	08	M	No	no	1m17d	NA	Hz (37.3%)	1y6m	2	N (100%)			
	p15	13	M	No	yes	1m7d	1	Partial (20%)	3m18d	1	Hz (38.6%)	11m14d	1	Hz (58.6%)
	p16	17	M	No	no	2m	1	Partial (25.3%)	2m16d	1	Hz (34.6%)	1y	2	Hz (40%)
	p17	19	F	NA	NR	NA	1	Profound (5.3%)	NA	1	Partial (17.3%)	NA	1	Partial (22.6%)
	p18	36	M	No	no	1m26d	1	Partial (21.3%)	NA	2	N (100%)			
	p19	38	M	No	NR	1m	1	Profound (2.6%)	3m10d	2	Partial (22.6%)	8m26d	2	Borderline Partial/Hz (29.3%)
**2b) from a less to a more severe category**	p20	8	M	NA	NR	1y22d	3	Hz (61.3%)	2y1m	1	Partial (20%)			
	p21	8	F	No	no	6m23d	1	Hz (42.6%)	2y1m	4	Partial (26.6%)			
	p22	20	M	No	no	NA	2	Hz (33.3%)	NA	2	Partial (21.3%)			

Lab, laboratory; F, female; M, male; y, years; m, months; d, days; NR, not reported; NA, not available; N, normal; Hz, heterozygous. The following enzyme activity ranges (in nmol/min/mL) were used for the classification of the biochemical phenotype (percentage in relation to the mean value of normality): <0.75 (<10%, profound deficiency); 0.75–2.25 (10–30%, partial deficiency); 2.26–4.99 (30.1–66.5%, heterozygosity). Any value in the normal range (5.0–10) was considered as corresponding to 100% of the normal activity. Values within ± 0.1 of a cut-off point were classified as borderline.

### *BTD* variants

Thirty-nine different genotypes were found in the entire group of patients (Table 1). In addition to the variants found in our previous study [12], five known pathogenic variants, one synonymous variant (c.1284C>T or p.Y428Y) and three novel variants were identified in the 34 patients added to this study. The novel variants were c.1337T>C (p.L446P), c.1466A>G (p.N489S) and c.962G>A (p.W321*) in exon 4. The variants p.L446P and p.N489S were not found in the controls and were both considered damaging by PolyPhen-2 (scores of 1 and 0.85, respectively) and Mutation Taster (probability of 0.99 for both) but were tolerated by SIFT (scores of 0.1 and 0.22, respectively).

In the *BTD* promoter region, 5'UTR and exon 1 analyses, three variants in the promoter region were identified: c.-514C>T (rs41284037), c.-315A>G (rs2019160, not shown in Table 1) and c.-183G>A (rs2279841), with allele frequencies of 3.6% (allele T), 99.3% (allele G) and 7.3% (allele A), respectively. Except for one patient (genotype 5 in Table 1, p7 in Table 3) who was heterozygous for the variant c.-315A>G, all other patients were homozygous for allele G.

Among known pathogenic variants, the most frequent were c.1330G>C (p.D444H), c.755A>G (p.D252G) and c.[1330G>C;511G>A] (p.[D444H;A171T]), with allele frequencies of 46.7%, 5.8% and 3.6%, respectively. The frequency of p.D444H (not considering this variant in complex alleles) tended to decrease in patients from the South (51.2%) to the Southeast (44.8%) and Northeast regions of the country (35.7%), although these differences were not statistically significant (exact p-value = 0.38).

Since 11 patients had variants of unknown phase (genotypes 30–33, Table 1) and/or variants not described previously (genotypes 34–39, Table 1), the expected biochemical phenotype could be determined for only 61/72 patients. Based on genotype, four patients (6.5%) were expected to present profound BD, 19 (31.1%) to present partial BD, 23 (37.7%) to present heterozygous activity (of these, 16 were homozygous for p.D444H), and 15 (24.5%) to present normal activity (of these, nine were heterozygous for p.D444H). Among patients classified as having profound BD, three were from the South and one was from Southeast Brazil. The frequency of patients classified as partial BD according to genotype was not significantly different among the South (42.9%), the Southeast (21.4%) and the Northeast regions (8.3%) (exact p-value = 0.058).

### Association between genotype and biochemical phenotype (Table 1)

Patients with no *BTD* variants or with only polymorphic variants (genotypes 01–03) were found among those patients who had lower than normal biotinidase activity, in the range for heterozygotes. Most patients heterozygous for p.D444H (genotypes 05–07) and homozygous for p.D444H (genotype 08) possessed activity within the heterozygous range. The majority of patients who were compound heterozygous for p.D444H and previously described severe variants (genotypes 16–25) had enzyme activity consistent with partial BD, except for five with heterozygous levels. The recurrent genotypes associated with different biochemical phenotypes are shown in S1 Fig (genotypes 05, 08, 17 and 22).

Among the novel genotypes, the patient who presented heterozygosity of the variant p.W321* (genotype 34) exhibited 56% activity (heterozygous range). An enzyme activity measurement was not available for the patient’s father, who had the same variant. A patient with the p.L446P variant in one allele and the normal sequence in the other (genotype 36, p18) had enzyme activity of 21.3% (partial BD) and in a second sample 100% (normal); his father was also heterozygous for the novel variant and exhibited activity of 100% (normal). The patient who possessed the p.N489S variant also possessed a known pathogenic variant in *trans* (genotype 39, p11) and had enzyme activity of 9.3% (borderline profound/partial BD) and, in a second assay, 18.7% (partial BD); the patient’s father, who was heterozygous for p.N489S, had enzyme activity of 53.3% (heterozygous range).

Comparisons between the expected and observed biochemical phenotype were possible for 54/72 patients, and concordance was observed in 37 (68.5%) cases. The clinical information for patients who were discordant is shown in Table 2. No discordant patients were premature, and neonatal jaundice was reported in only one patient from this group (dp1). Among the patients with an observed biochemical phenotype worse than expected, the biotinidase activity test was performed in a state other than the state of residency for 9/11, and so we assume the plasma samples for these patients underwent a prolonged period of transport prior to the enzyme test being performed. For 5/6 patients with an observed biochemical phenotype better than expected, the biotinidase activity test was also performed in a state other than the state of residency and plasma samples likely also underwent a prolonged period of transport before the enzyme test was performed. Among patients expected to have biotinidase activity in the normal range, patients dp1-dp4 and dp6-dp11 exhibited enzyme activity suggestive of heterozygosity, and dp5 exhibited enzyme activity suggestive of partial BD. Patients dp3 and dp4 showed the c.-514C>T variant, patient dp7 was the only heterozygous patient for c.-315A>G (allele A was in *trans* with the other variant presented), and patient dp10 had the non-coding variant c.-183G>A. Conversely, patient dp12 exhibited normal enzyme activity, and patient dp13-17 showed levels suggestive of heterozygosity (considering the highest result), although their genotypes suggested heterozygous levels and partial BD, respectively. Patient dp17 exhibited the non-coding c.-183G>A variant. Among the 17 discordant patients, the c.-183G>A, c.-315A>G and c.-514C>T variants were present in heterozygosis in two (12%), one (6%) and two (12%) patients, respectively. The c.-183G>A and c.-514C>T variants were also present in eight (22%) and two (5%) of 37 concordant patients, respectively.Patients with borderline biotinidase activity had an expected biochemical phenotype equal to the higher classification. Among patients whose classification changed between consecutive tests (Table 3), the expected biochemical phenotype matched the worst result for 3/9 patients and the best result for 6/9 patients.

### *In silico* evaluation of non-coding variants

The c.-514C>T, c.-315A>G and c.-183G>A variants map to an evolutionarily conserved region in chimpanzee (1,002 bp) and rhesus monkey (3,212 bp), and c.-514C>T is also conserved in dog (228 bp), mouse (444 bp) and rat (221 bp). S2 Fig shows that the cytosine at position -514 is not conserved in rat; at position -315, there is an adenine or guanine; and at position -183, guanine is not conserved in mouse and rat.

According to *in silico* prediction, the c.-514C>T variant is located in an Sp1 transcription factor (Sp1) binding site, and the T allele creates one more site for Sp1 and two for neurofibromin 1 (NF-1) (S3A Fig). In the c.-315A>G variant, no binding sites for human transcription factors are predicted, independent of the allele. Finally, allele A of the c.-183G>A variant is predicted to change the site for CCAAT/enhancer binding protein alpha (C/EBPalpha) to a site for CCAAT/enhancer binding protein delta (C/EBPdelta) and to create a site for Sp1 (S3B Fig). The *BTD* gene and the aforementioned transcription factor genes exhibit mRNA expression in most of the tissues assessed in the studies in Gene Cards database. Protein expression differed, however, with NF-1 found in liver and pancreas, C/EBPalpha detected in the liver, and C/EBPdelta and Sp1 present in the pancreas. The transcription factors Sp1 and C/EBPdelta were included in the list of transcription factors identified in the 5’ region of the *BTD* gene by the ENCODE ChIP-Seq Significance Tool.

## Discussion

Currently, BD is included in many National Neonatal Screening Programs, including the USA, Canada, the Netherlands and the United Arab Emirates [13, 23–25], with the primary goal of identifying profound BD. Screening is performed through measurement of biotinidase activity in dried blood spot samples. There is some controversy regarding the indication of treatment for partial BD patients, but currently, in essentially all programs, it is recommended that these children be treated with biotin [26]. In the literature, there is no consensus regarding the stage at which *BTD* gene analysis should be performed. This is in part due to misconceptions about the linearity of the genotype-phenotype relationship and predictions. In addition, the necessity of determining the *cis/trans* status of the mutations identified in patients is usually not stressed.

In Brazil, BD has been included in the National Neonatal Screening Program since 2012, and the classifications of profound BD, partial DB, and heterozygous activity are applied to test results. This classification is directly related to therapeutic decision-making: profound and partial BD patients are given oral biotin supplements, but individuals with activities in the heterozygous range are not. The labelling of asymptomatic individuals who present with less than 30% residual biotinidase activity as patients and prescribing life-long treatment is an issue that concerns health professionals. Our data suggest that in Brazil, neonatal screening is identifying mainly individuals with partial DB and heterozygous activity and that biotinidase activity is not always successful in differentiating between the different disease severities.

### Genotype-biochemical phenotype association

In our study, the genotype-biochemical phenotype association is not always consistent. For example, some patients who presented with the same genotype had different biochemical phenotypes. Patient dp12 (genotype 08), who presented with normal activity despite homozygosity for D444H, is an interesting case, having only been described once before [23].

Neonatal jaundice, prematurity and transportation conditions of collected sample do not appear to be the main causes of the discordance between the observed and expected biochemical phenotypes because none of the discordant patients was premature, and only one discordant patient experienced jaundice. In addition, for patients with biotinidase activity that was both worse and better than expected, the biotinidase activity test had been performed on samples that underwent a period of transport to another state before analysis.

The variants we identified in the promoter region of *BTD* do not seem to have a relevant effect on biotinidase activity. However, it cannot be excluded that the variant c.-514C>T contributes to a subtle decrease in gene expression since patients without pathogenic mutations (dp3 and dp4) and heterozygous for this variant have activities consistent with heterozygosity. The c.-315A>G (allele A) might also have a subtle decreasing effect since it was only present in a discordant patient who presented with an activity worse than expected (dp7).

The bioinformatics analysis suggested that the c.-514C>T variant is located in a more evolutionarily conserved region than the other variants and that the c.-514C>T and c.-183G>A variants might be critical for transcription factor binding. The co-expression of biotinidase and transcription factors predicted to have binding sites that are created or abolished by these variants reinforces the likelihood of these variants affecting *BTD* gene expression, but further studies are needed.

### Non-genetic modifier factors

Our data on consecutive tests performed on the same patient suggest that enzyme activity increases with age, in particular during the neonatal period. In addition, prematurity and neonatal jaundice might have contributed to the first lower results. Schulpis et al. [5] suggest that the reduced biotinidase activity in jaundiced babies may be due to impaired liver function and that high concentrations of total bilirubin in plasma may act as an inhibitor of biotinidase.

Our data does not support the concept that male babies have lower biotinidase activity due to a higher degree of hepatic immaturity since the majority of patients whose condition changed to a better or to a worse classification were male. It is important to note that the comparison of consecutive measurements could be biased since the reason for performing a second test was not always clear, and consecutive tests were not always performed by the same laboratory.

### Genetic data

The *BTD* gene variants p.D444H, p.D252G and p.[D444H;A171T] accounted for 56% of the known pathogenic alleles in this study. These variants were also among the most frequent identified in the Brazilian sample evaluated by Neto et al. [10] (n = 21). The frequencies of p.D444H, partial BD and partial BD according to genotype tend to decrease from the South to the Southeast and from the Southeast to the Northeast. Swango et al. [27] observed p.D444H more frequently in patients whose parents were of German descent, suggesting a potential founder effect. According to “The 1000 Genomes Project (Phase 3)”, the p.D444H allele frequency differs among populations, with 4% prevalence in European populations, to 2–3% in South Asian and American populations (excluding Brazilians) and zero in African and East Asian populations. European ancestry is the major contributor to the genetic background of Brazilians (followed by African and Amerindian ancestries), and the South has a greater European contribution than the Southeast and Northeast [28]. These geographical differences provide a likely explanation for the apparent difference in p.D444H frequencies observed in Brazil. The frequency of p.D444H in the Brazilian population from the South is 4% [12], but similar data are not available for the populations of other regions.

The novel variants p.L446P and p.N489S do not appear to be polymorphic in the Brazilian population. The *in silico* pathogenicity predictions for the variant p.L446P are divergent, but based on the enzyme activity exhibited by the patient and his father, this variant appears to be non-pathogenic. The variant p.N489S is located at a potential N-linked glycosylation site [29] and could be considered mildly pathogenic, similar to p.D444H, according to the patient’s highest activity (partial BD), although the patient’s first biotinidase activity measurement was compatible with profound BD, and the activity shown by the patient’s father was at the level of heterozygosity. Previously, another nucleotide substitution in the same position, c.1466A>C (p.N489T), was reported and considered pathogenic [30]. The novel variant p.W321* creates a stop codon at the end of the nitrilase/amidase homologous domain (which reaches amino acid 367), and thus this variant must be considered pathogenic.

Exon 1 contains the first translation start site and encodes the peptide signal. Some groups have analysed the initial portion of the *BTD* gene in patients with BD [31–34]. These studies observed a point variant in the promoter [32] and another in the 5’UTR [35]. In addition, a 107-kb contiguous deletion including the promoter, exon 1 and a portion of intron 1 of the *BTD* gene, the entire *HACL1* gene and a small portion of the of the *COLQ* gene [36] have been reported. Previous studies in Brazil have not identified variants in the 5'UTR and exon 1 [9, 10], and variants in these regions were not observed in the larger and more diverse sample examined in the present study. This is the first study to sequence the *BTD* promoter in Brazilian patients with reduced biotinidase activity, and the c.-514C>T, c.-315A>G and c.-183G>A variants were identified and are considered polymorphic based on their frequencies of 6% (allele T), 96% (allele G) and 17% (allele A) in “The 1000 Genomes Project (Phase 3)”, respectively.

## Conclusion

Our data suggest that biotinidase activity increases with age and that prematurity and neonatal jaundice might decrease biotinidase activity. The expected and observed biochemical phenotypes agreed in nearly 70% of cases. Neonatal jaundice, prematurity, transportation of the sample and non-coding variants do not appear to be the main causes of the discordance between the genotype and biochemical phenotype in our study. The disparity between the genotype and biochemical phenotype reinforces the idea that BD should be diagnosed using biotinidase activity. Since enzyme activity can be influenced by various factors, we recommend performing more than one biotinidase activity measurement, preferably in the same laboratory, with at least one sample collected after the neonatal period, and the highest enzyme activity result should be used for classification. In the context of BD treatment, DNA analysis of the patient and parents may be useful after the second biotinidase activity measurement if the result is approximately 30% of normal activity to gather further evidence for partial BD and consequent treatment intervention.

## Supporting information

S1 Fig Biotinidase activity of recurrent genotypes associated with different biochemical classifications. Profound Deficiency–biotinidase activity <10%, Partial Activity–biotinidase activity 10–30%, Heterozygous–biotinidase activity 30.1–66.5%. Any value in the normal range (5.0–10 nmol/min/mL) was considered 100% of the normal activity.
(TIF)

S2 Fig Multiple alignment of the human (Homo sapiens), chimpanzee (*Pan troglodytes*), rhesus monkey (*Macaca mulatta*), mouse (*Mus musculus*) and rat (*Rattus norvegicus*) *BTD* gene sequences. A, B and C show the positions of the non-coding c.-514C>T, c.-315A>G and c.-183G>A variants, respectively.
(TIF)

S3 Fig **Output of the prediction tool for transcription factor binding sites (Alibaba) for the region of the variants c.-514C>T (A) and c.-183G>A (B).** * The transcription factor REB1 is not a human protein.
(TIF)

## Abbreviations

UTR: untranslated region
BD: biotinidase deficiency
ChIP-Seq: chromatin immunoprecipitation sequencing
Sp1: Sp1 transcription factor
NF-1: neurofibromin 1
C/EBPalpha: CCAAT/enhancer binding protein alpha
C/EBPdel: CCAAT/enhancer binding protein delta
