# Correction: Biotinidase deficiency: Genotype-biochemical phenotype association in Brazilian patients

**DOI:** 10.1371/journal.pone.0180463

**Published:** 2017-06-22

**Authors:** Taciane Borsatto, Fernanda Sperb-Ludwig, Samyra E. Lima, Maria R. S. Carvalho, Pablo A. S. Fonseca, José S. Camelo, Erlane M. Ribeiro, Paula F. V. de Medeiros, Charles M. Lourenço, Carolina F. M. de Souza, Raquel Boy, Têmis M. Félix, Camila M. Bittar, Louise L. C. Pinto, Eurico C. Neto, Henk J. Blom, Ida V. D. Schwartz

Several authors’ names appear incorrectly in the citation. The correct citation is: Borsatto T, Sperb-Ludwig F, Lima SE, Carvalho MRS, Fonseca PAS, Camelo Jr JS, et al. (2017) Biotinidase deficiency: Genotype-biochemical phenotype association in Brazilian patients. PLoS ONE 12(5): e0177503. https://doi.org/10.1371/journal.pone.0177503
