# Supplementary figures and images for: Biotinidase deficiency: Genotype-biochemical phenotype association in Brazilian patients
Source: PLoS One. 2017 May 12;12(5):e0177503. doi: 10.1371/journal.pone.0177503 (PMC5428951; doi:10.1371/journal.pone.0177503)

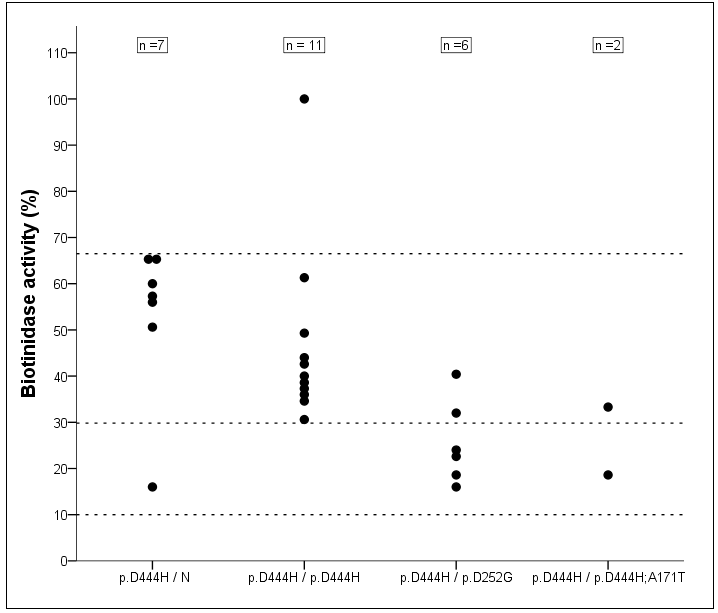

Supplement: S1 Fig — Profound Deficiency–biotinidase activity <10%, Partial Activity–biotinidase activity 10–30%, Heterozygous–biotinidase activity 30.1–66.5%. Any value in the normal range (5.0–10 nmol/min/mL) was considered 100% of the normal activity. (TIF) [file pone.0177503.s001.tif]

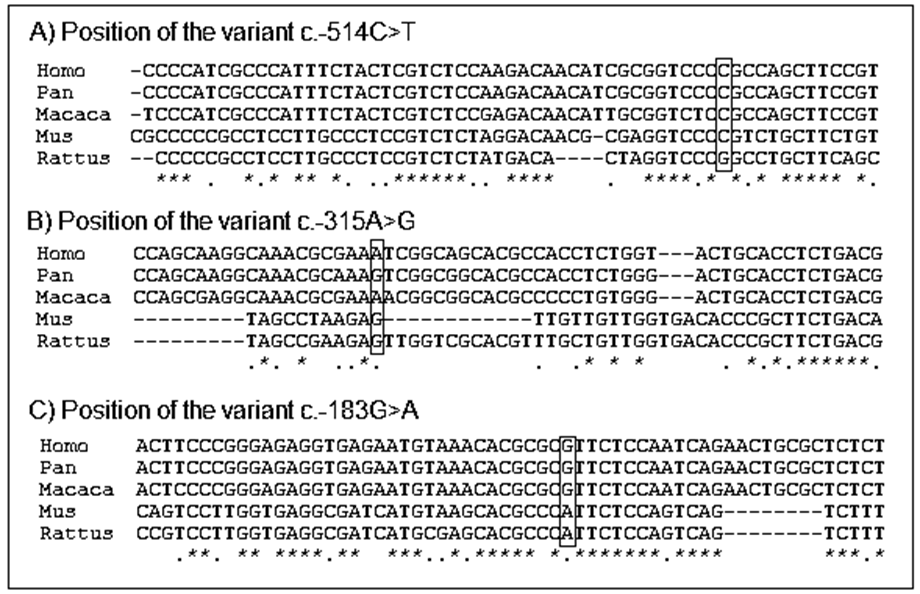

Supplement: S2 Fig — A, B and C show the positions of the non-coding c.-514C>T, c.-315A>G and c.-183G>A variants, respectively. (TIF) [file pone.0177503.s002.tif]

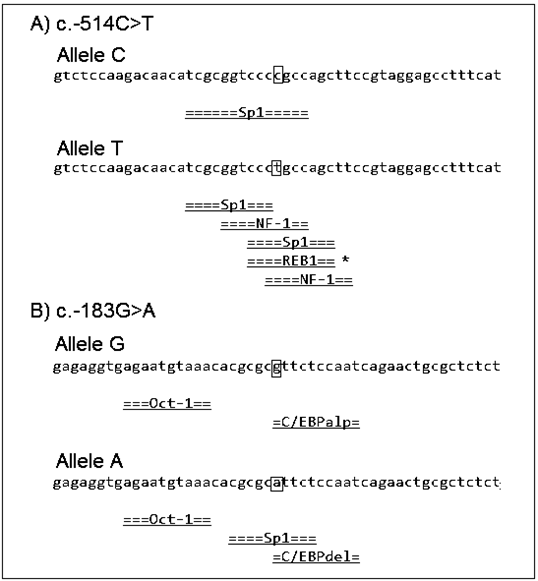

Supplement: S3 Fig — Output of the prediction tool for transcription factor binding sites (Alibaba) for the region of the variants c.-514C>T (A) and c.-183G>A (B). * The transcription factor REB1 is not a human protein. (TIF) [file pone.0177503.s003.tif]
